# Supplementary material for: Phage-plasmids promote recombination and emergence of phages and plasmids
Source: Nat Commun. 2024 Feb 20;15:1545. doi: 10.1038/s41467-024-45757-3 (PMC10879196; doi:10.1038/s41467-024-45757-3)
Supplement: Supplementary file 1 — Supplementary Information [file 41467_2024_45757_MOESM1_ESM.pdf]

Supplementary Information

Phage-plasmids promote recombination and emergence of phages and plasmids

Authors:
Eugen Pfeifer[1]*, Eduardo P.C. Rocha[1]*

[1] Institut Pasteur, Université Paris Cité, CNRS UMR3525, Microbial Evolutionary Genomics, Paris 75015, France

*corresponding authors: eugen.pfeifer@inrae.fr, erocha@pasteur.fr

Running title
Gene flow and fate of phage-plasmids

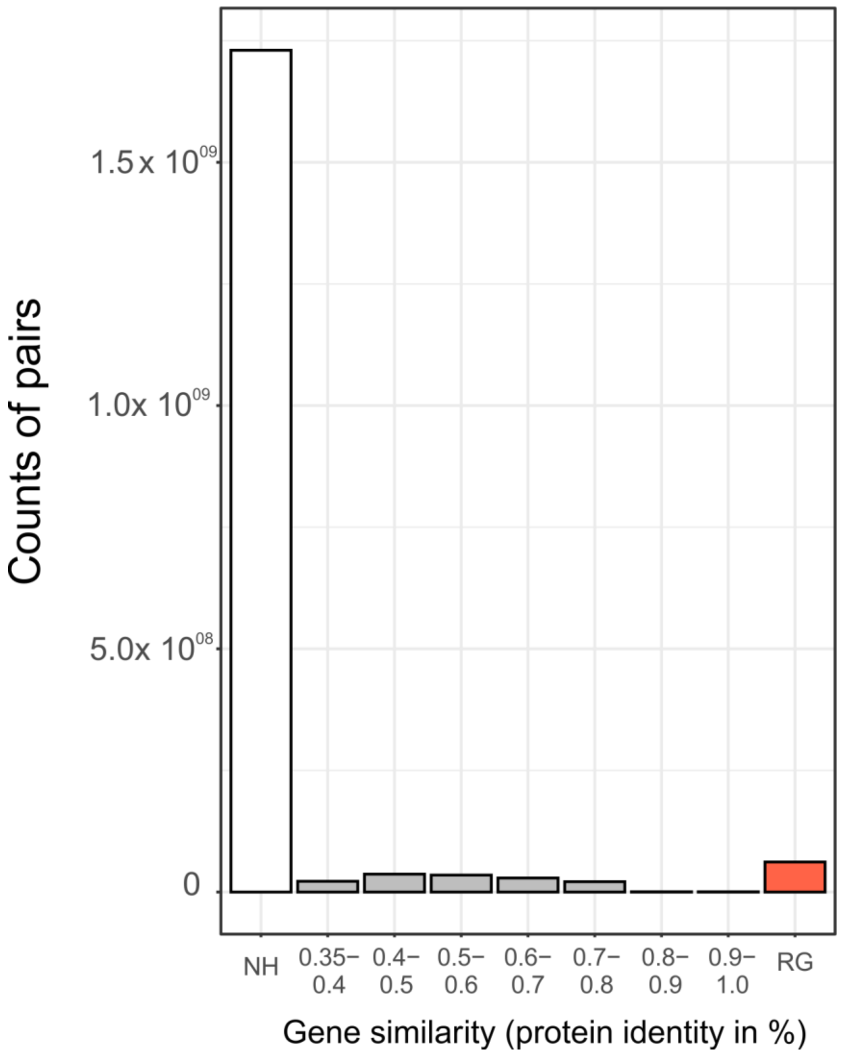

Figure S1: Distribution of sequence similarity between homologs in pairs of recombining mobile genetic elements. Homologous and non-homologous sequences were quantified between all plasmids, phages and phage-plasmids having a wGRR <0.1. Protein sequence identity of pairs of homologs was binned between 35% and 100%. Gene pairs with >80% identity and covering >80% of the alignment were assigned as RGs. The number of non-homologs (NH) between pairs of MGEs was computed by taking the mean (per pair) of the number of dissimilar genes. For example, if MGE A (18 genes) and MGE B (20 genes) share 2 homologous genes, then mean(AB) of NHs = (18-2 + 20-2)/2 = 17.

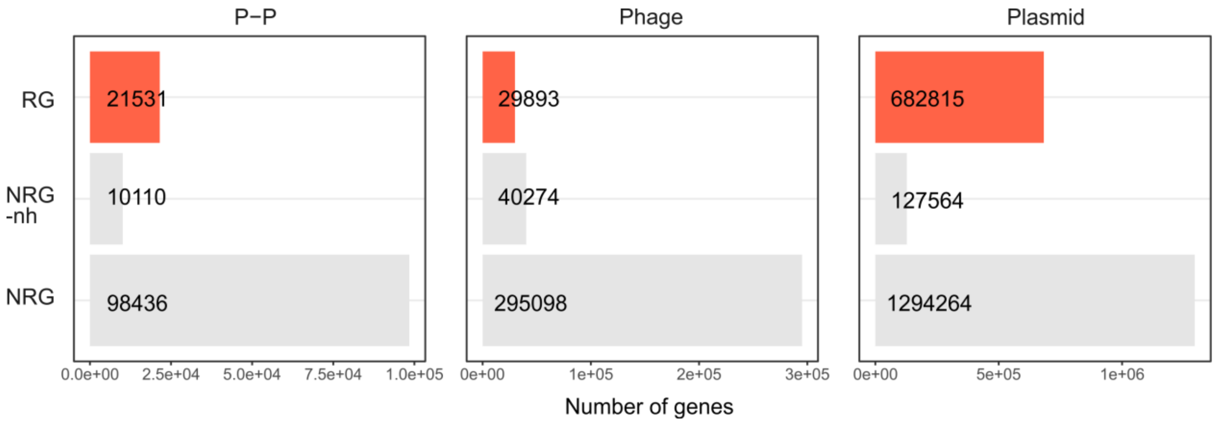

Figure S2: Counts of recombining genes (RG), non-recombining genes (NRGs) and NRGs with no homologs (NRG-nh) in phages, plasmids and phage-plasmids. Numbers represent absolute counts.

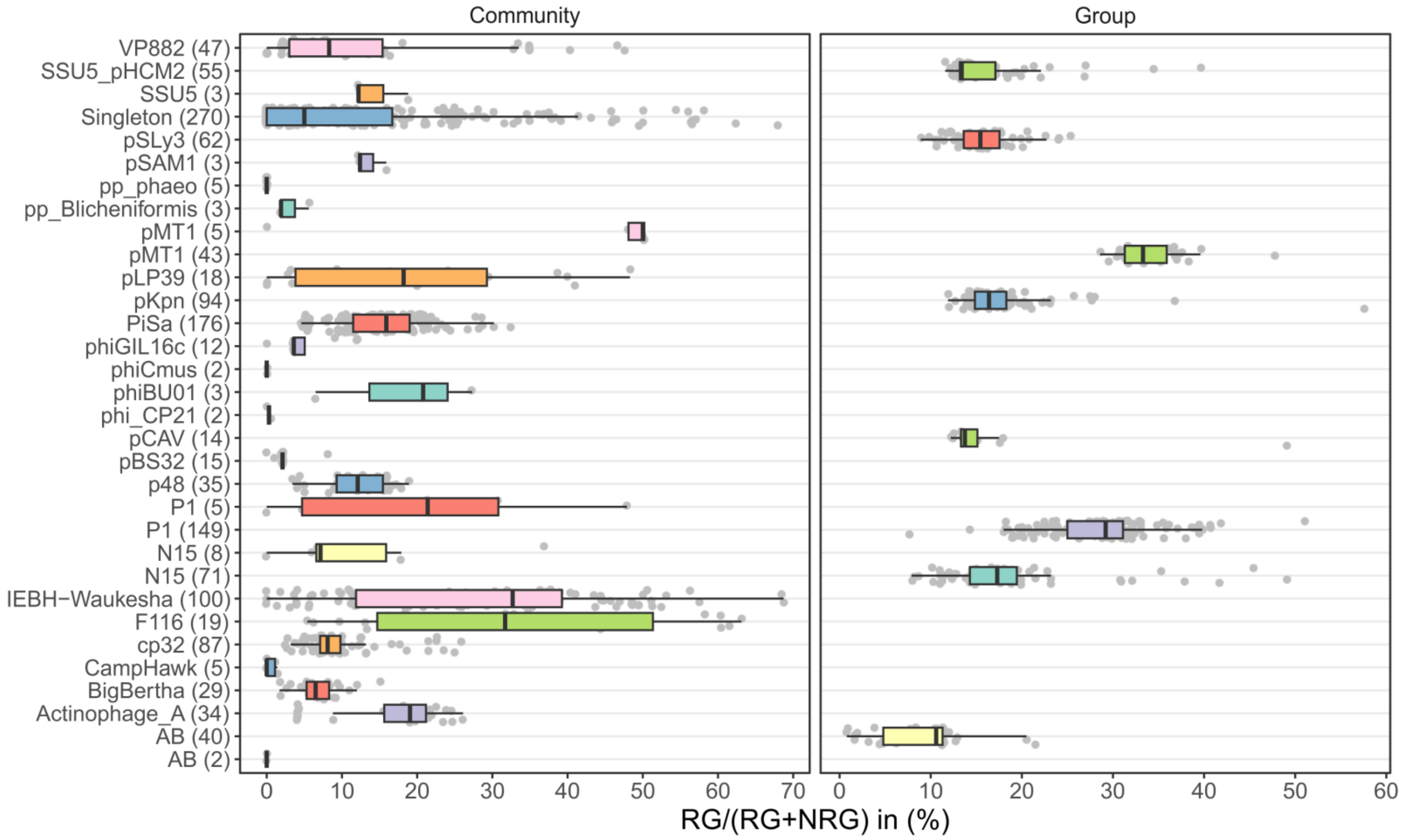

Figure S3. Recombining genes (RGs) per genome for phage-plasmids (P-Ps) of different groups. Each dot is representing a fraction of RGs per P-P genome (RG Fraction = RG/ (RG+non-RG))). P-Ps were grouped in well-related groups (right panel) and diverse communities (left panel). Numbers in brackets represent the numbers of members within groups and communities. Groups were curated out from communities as done in our previous work[13]. Singletons were pooled together and are shown in the community panel. In box plots, whiskers' lengths were set to 1.5 times the interquartile range (default setting in ggplot2 (https://ggplot2.tidyverse.org/reference/geom_boxplot.html)

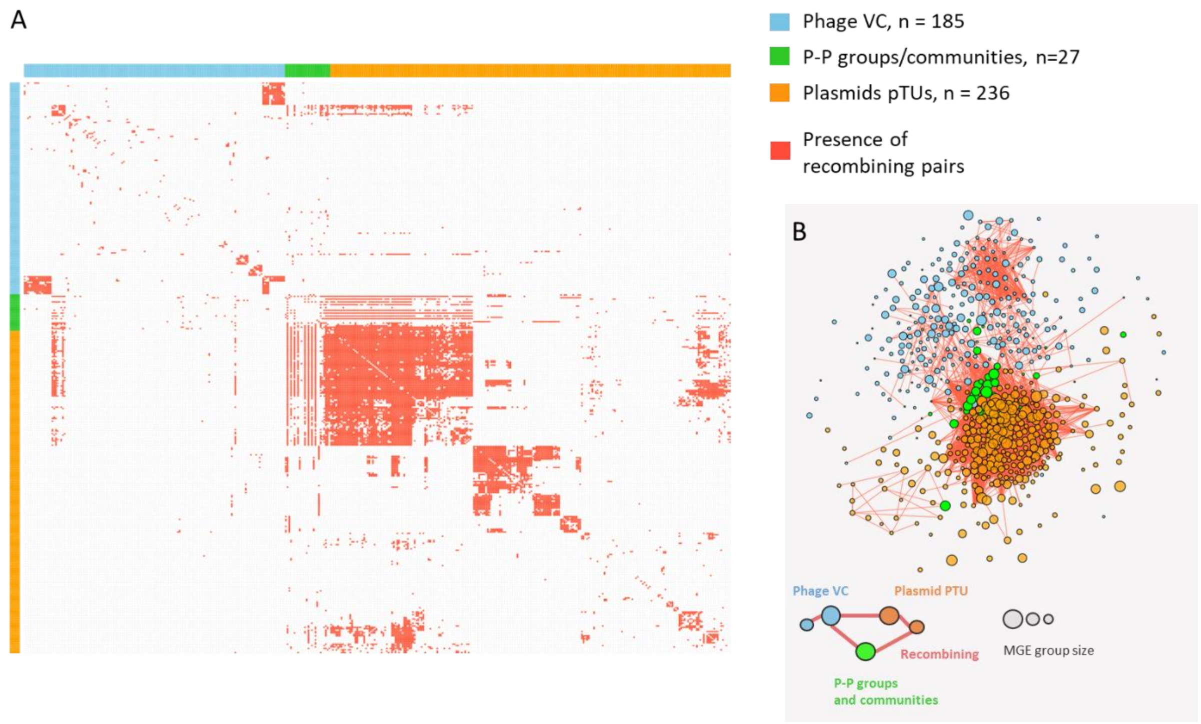

Figure S4. Network of weighted gene repertoire relatedness (wGRR) between groups of phages, plasmids and phage-plasmids**. A.** Heatmap indicating the comparisons between groups of phage (blue ribbon), P-Ps (green) and plasmids (orange). Mobile genetic elements are organized in types, and all groups within types were ordered using hierarchical clustering. A recombination event is highlighted in red, if there is at least one recombining pair between the corresponding groups of mobile elements. **B.** Same graph as in Figure 1. Edges highlight paths of gene exchange.

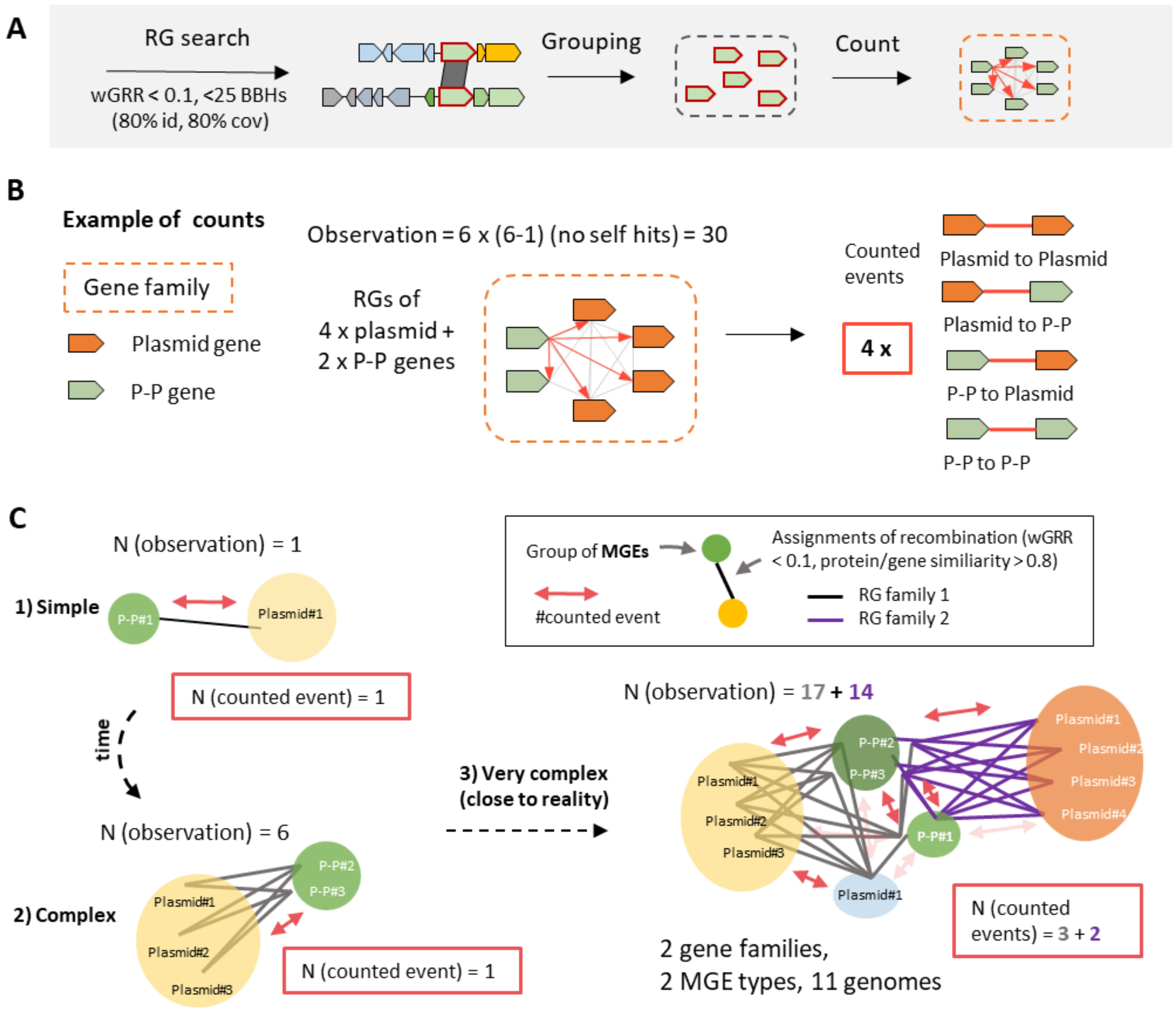

Figure S5. Quantitative assessment of recombining genes between phages, plasmids and P-Ps. **A.** RGs were identified as described in Methods. These genes were grouped into families using single-linkage clustering (at least 80% sequence identity and 80% coverage of the alignment on the genes). Recombining gene families (RG families) were counted according to the recombining pair assignment to phages, plasmids and/or P-Ps (Figure 2B). **B+C.** Examples of the way we counted the events of exchange. **B.** If an RG family consists of four genes from plasmids and two genes from P-Ps, we observe overall 30 events (self-hits excluded) since each gene has an assignment to the five other genes (grey lines, one example highlighted in red arrows). To avoid inflating the number, we count in this case up to four transfers: Plasmids <-> Plasmids, P-Ps <-> P-Ps, P-Ps <-> Plasmids, Plasmids <-> P-Ps. **C.** 1) If a P-P shares a RG with a plasmid, we counted it as one event. 2) However, very similar elements that share RGs with other elements (that form a group) may cause a huge amplification of RG assignments, while the recombination event may have occurred in the recent past only one or a few times. 3) Different RG families have different counts (different histories) depending on the MGE types. To avoid counting the same event many times, we classed RGs in families and counted only the presence of the families in different MGE types.

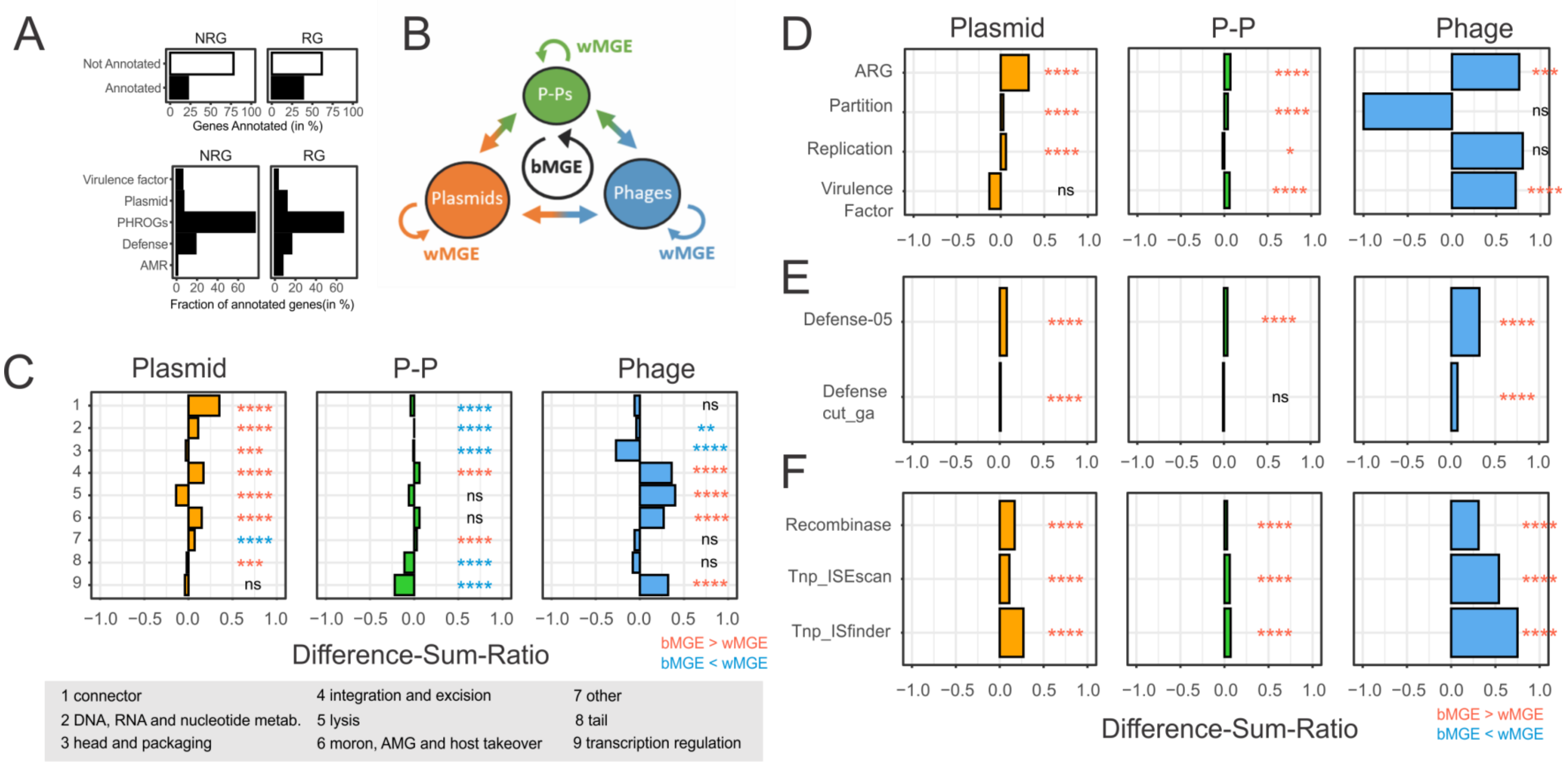

Figure S6: Gene flow between mobile elements in terms of gene function. A. All recombining genes (RG) and non-RGs (NRGs) were annotated by various databases (see Methods, see Supplementary Dataset S3). Shown are fractions of annotated RGs and NRGs. **B.** RGs were categorized in two groups: 1) Genes involved in exchanges between different types of MGEs (bMGE, inter MGE) and within the same type of MGE (wMGE, intra MGE). **C-F.** Enrichment and depletion tests were done as described for Figure 3 for genes matching bMGEs vs wMGEs (instead of RGs vs NRGs). All numbers of genes in the categories, and the adjusted p-values are listed in Supplementary Dataset S6. Shown are the Diff-Sum-Ratio calculated for genes matching bMGEs in the functional categories **(C)** given by PHROGs, **(D)** AMRFinderPlus, VFDB and plasmid-specific profiles, **(E)** DefenseFinder models (including the search with 50% profile coverage and domain ievalue $<1e^{-3}$, Defense-05), and **(F)** transposases and recombinases.

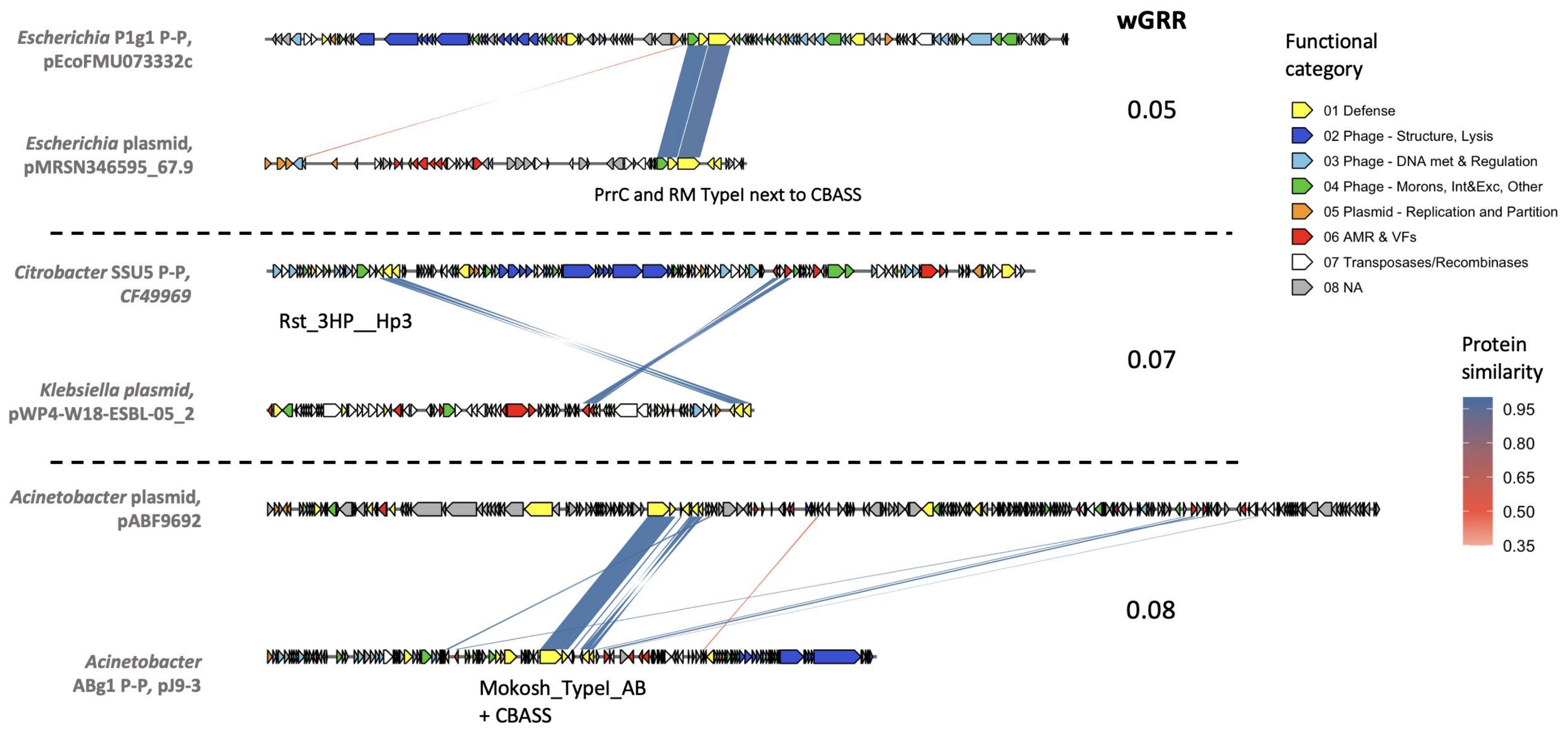

Figure S7. Exchanges of complete defense systems between P-Ps and plasmids. Examples of dissimilar plasmids and P-Ps with highly similar complete defense systems. Top panel: NZ_CP017847 and NZ_CP018111, middle panel: NZ_CP070546 and NZ_AP022059, lower panel: NZ_CP048828 and NZ_CP041590. Protein similarity indicates percentage identity between two protein sequences. This and the following genome-to-genome (synteny) plots were generated using gggenomes (https://github.com/thackl/gggenomes).

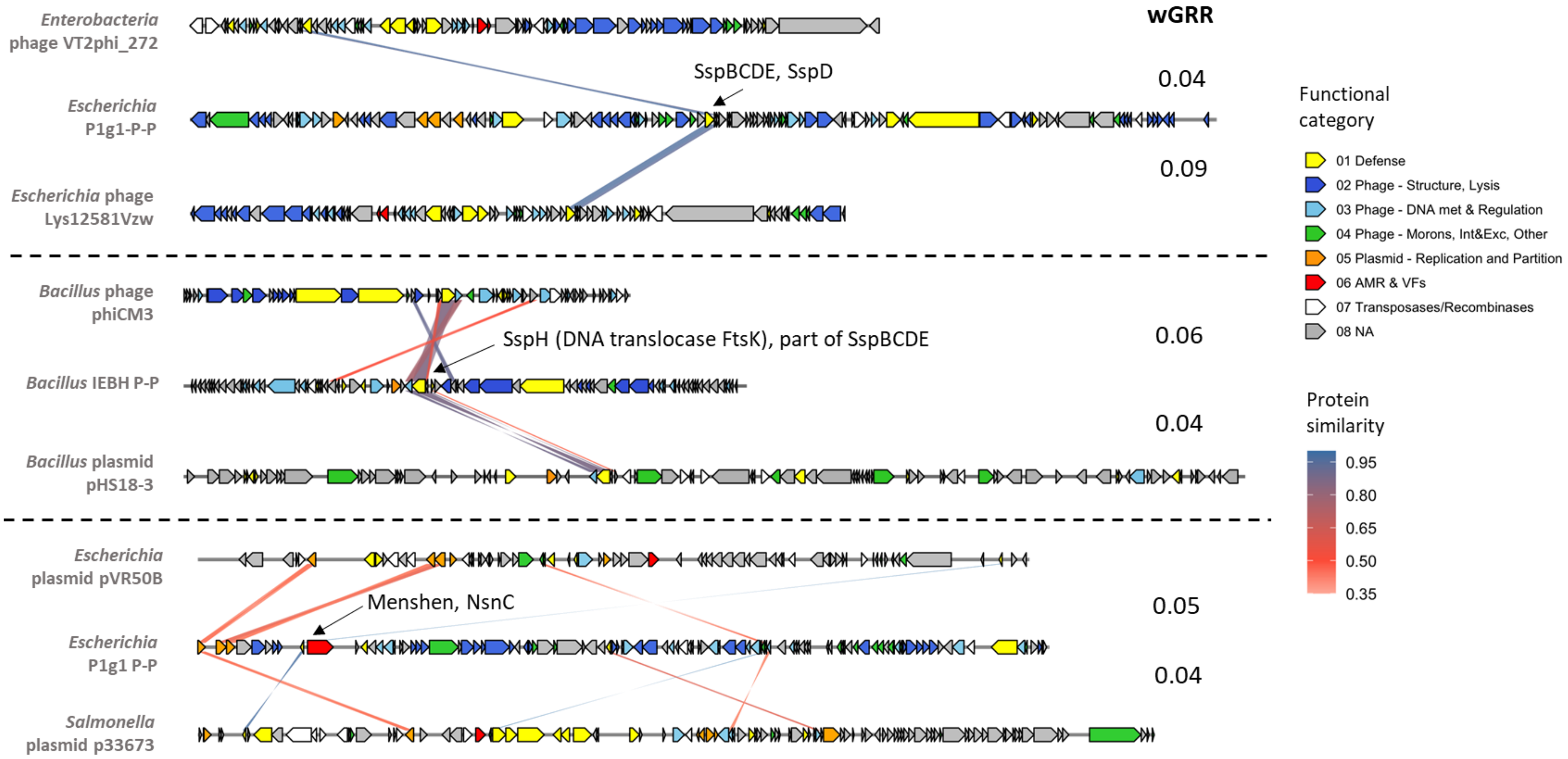

Figure S8. Recombining defense genes between P-Ps, phages and plasmids. As in Figure S7 but with examples of phages, plasmids and P-Ps having recombining defense genes (incomplete systems). Top panel: NC_028656, NZ_CP015837, NC_049917), middle panel: NC_023599, NZ_CP032613, NZ_CP012102, lower panel: NZ_CP011136, NZ_CP045829, NZ_CP012683.

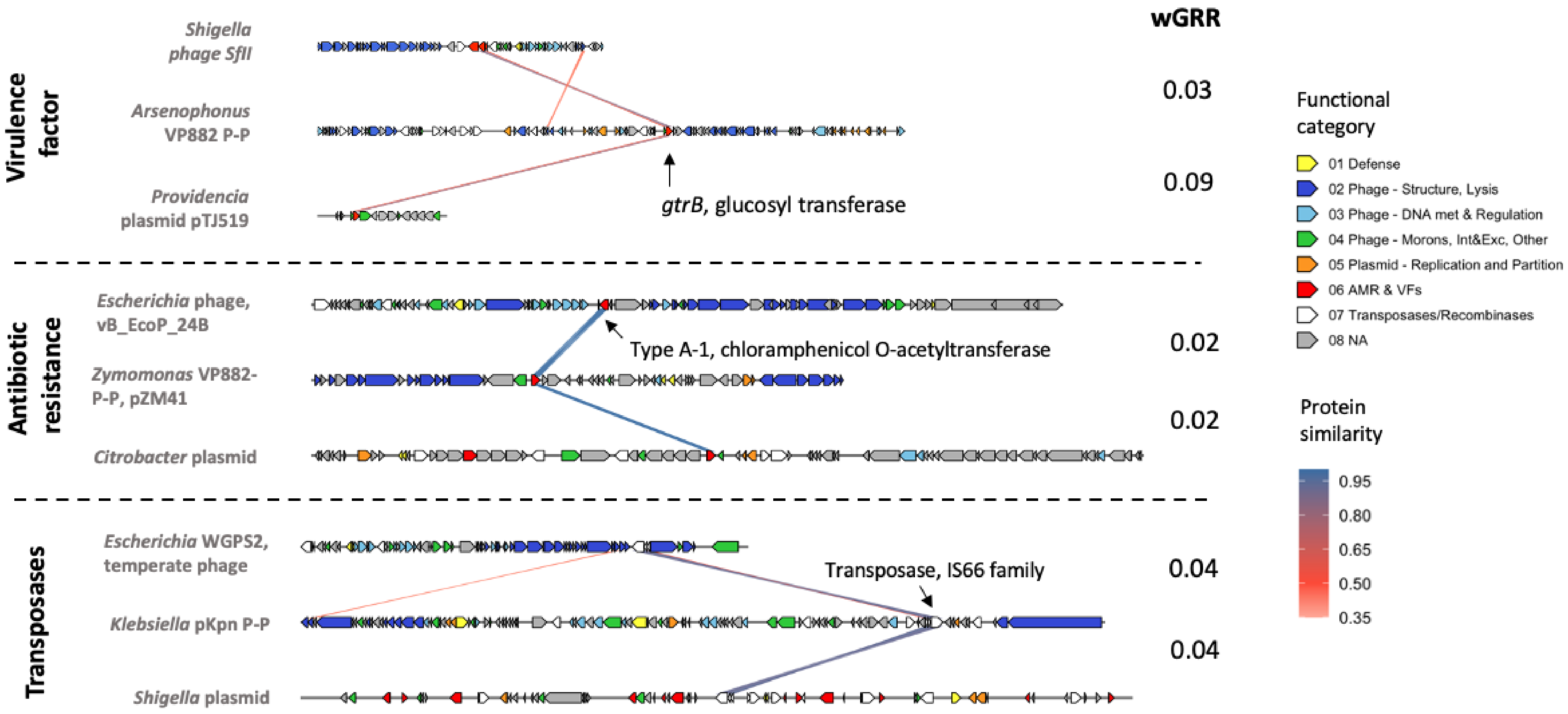

Figure S9. Examples of recent recombination events between P-Ps, plasmids and phages. Pointed out are cases in which recent exchanges occurred between phages, P-Ps and plasmids (direction not clear). Top panel gives example of a virulence factor encoding a glycosyl transferase (NC_019927, NZ_CP038615, NZ_CP058959), middle panel shows an example where an ARG encoding a type A-1 chloramphenicol O-acetyltransferase (resistance to chloramphenicol) was acquired through recombination by a phage, P-P and plasmid (NC_027984, NZ_CP023686, NZ_CP029735). Bottom panel exhibit an example where the same transposases gene (of the IS66 family) is detected in a phage, P-P and plasmid (NC_049941, NZ_CP069855, NZ_CP041512).

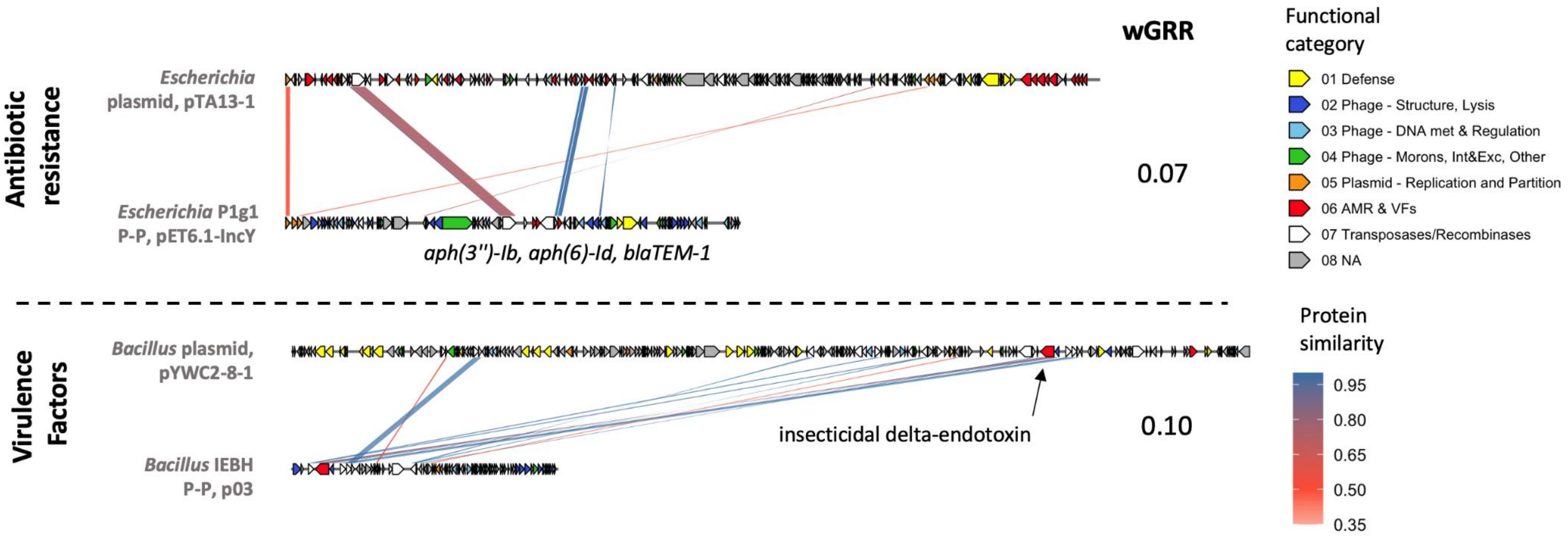

Figure S10. Recombination events between P-Ps and plasmids mediated by transposases/recombinases. Shown are examples of ARGs, (top panel), virulence factor (middle panel) and defense genes which transfer was likely facilitated by transposases or recombinases. Top panel ARGs: (NZ_CP069713 vs NZ_CP043218), middle panel virulence factors: (NZ_CP013056 vs NC_018487), bottom defense genes: (NZ_CP041590 vs NZ_CP050433).

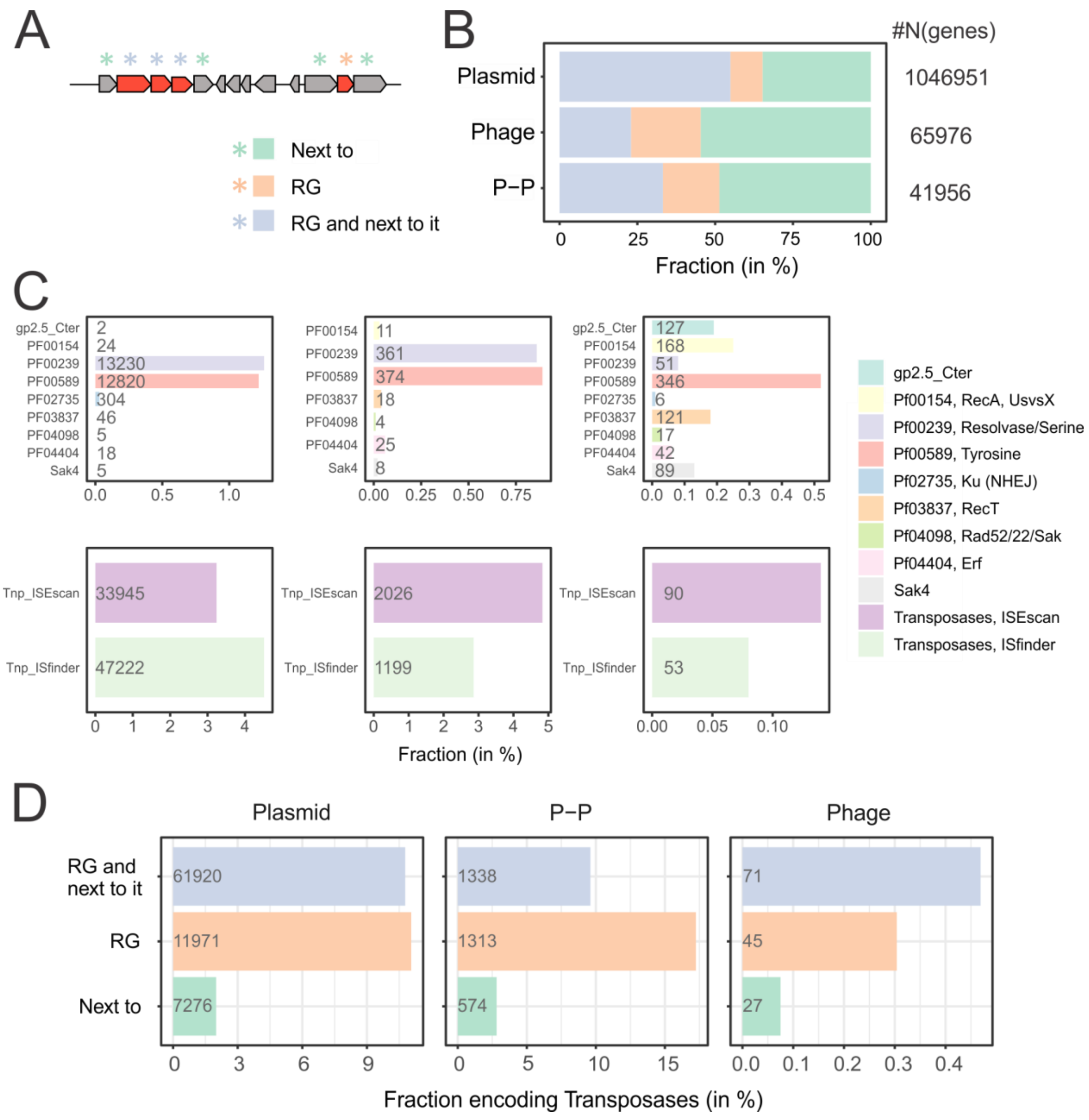

Figure S11. Transposases and recombinases were searched in RGs and RG-flanking genes. **A+B**. RGs and adjacent genes were allocated in groups of RGs-single (orange), genes proximal to RGs (green) and RGs that flanks other RGs (violet). **A**. Illustration how RG and gene neighbors are allocated and counted. Red genes represent RGs and stars indicate the counts with the group color. **B**. Distribution of RGs and their proximal genes in phages, plasmids and P-Ps. **C.** Same as in Figure 3, but direct neighbors of RGs were included. **D.** Fraction of transposases in RGs, genes flanking RGs and RGs that are adjacent to RGs (same color scheme as in A and B). Fraction of genes not encoding transposases are not shown. Numbers inside bars represent absolute counts of genes.

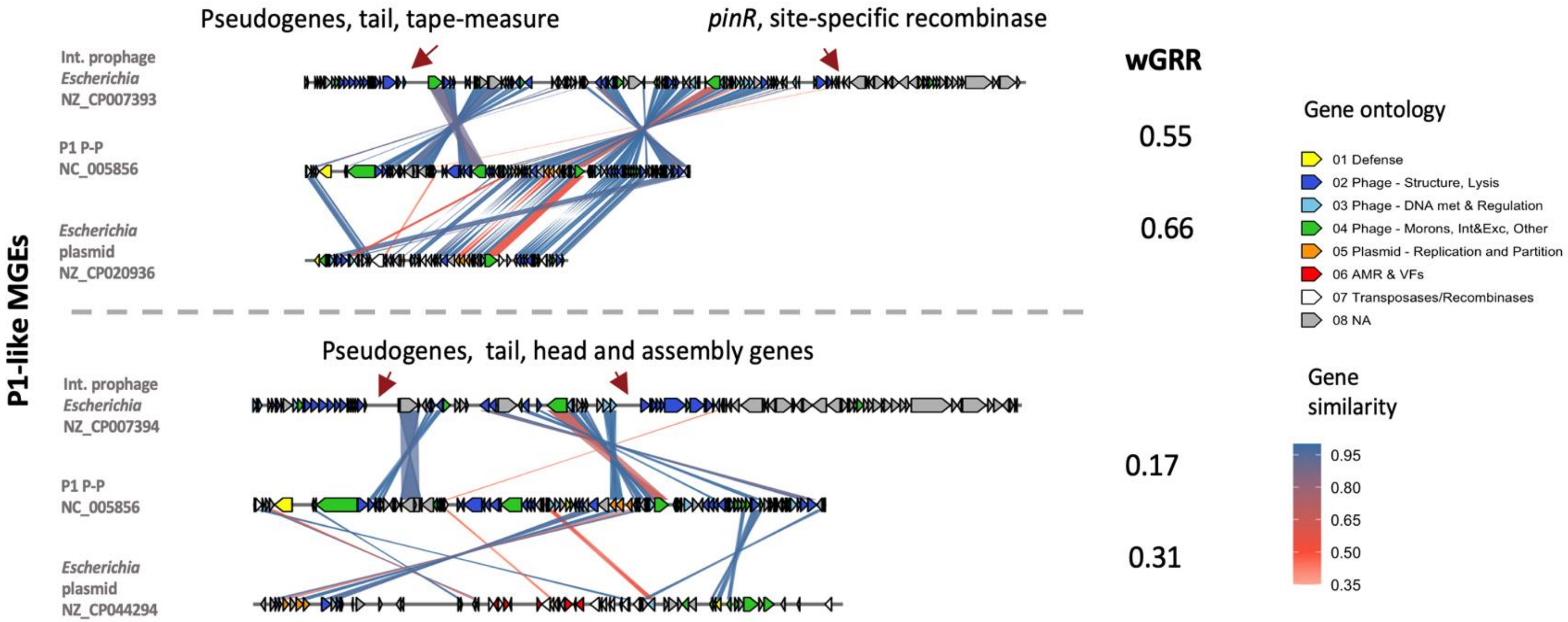

Figure S12. Synteny plot of P1 and P1-like plasmids and P1-like integrative prophages. Closely related examples are shown in the upper panel and less related examples are shown below.

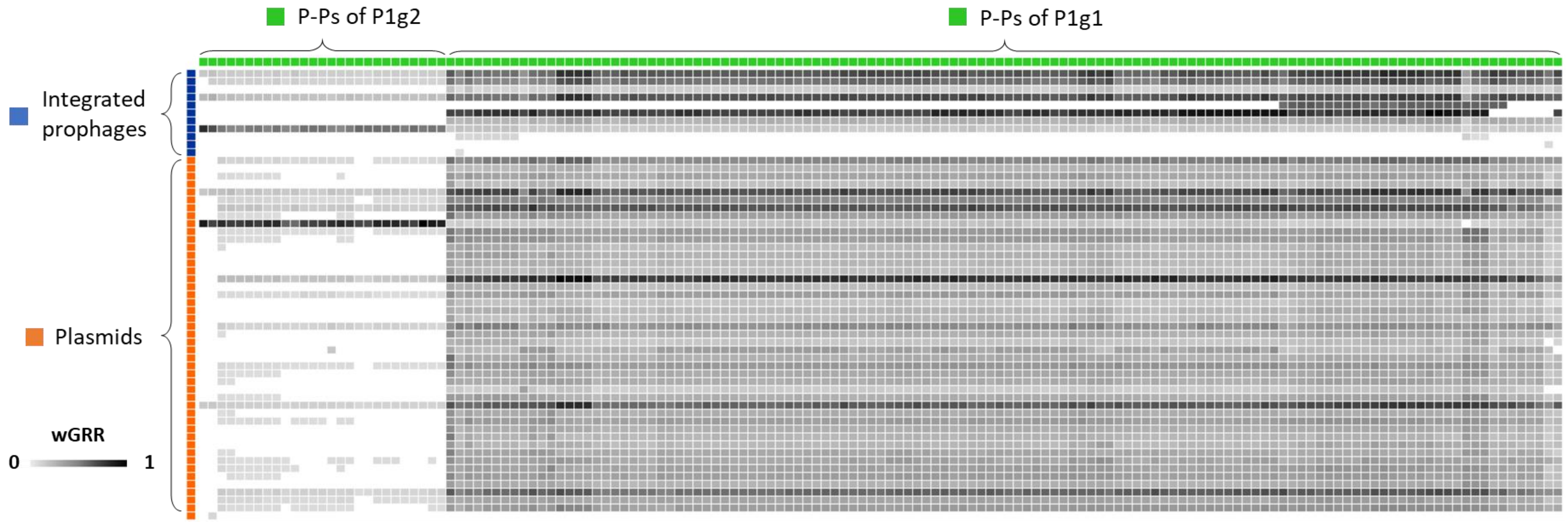

Figure S13: wGRR matrix of P1-like P-Ps and related plasmids and integrative prophages. Integrated prophages are shown in blue, plasmids in orange and P-Ps in green. The squares represent wGRR values between elements.

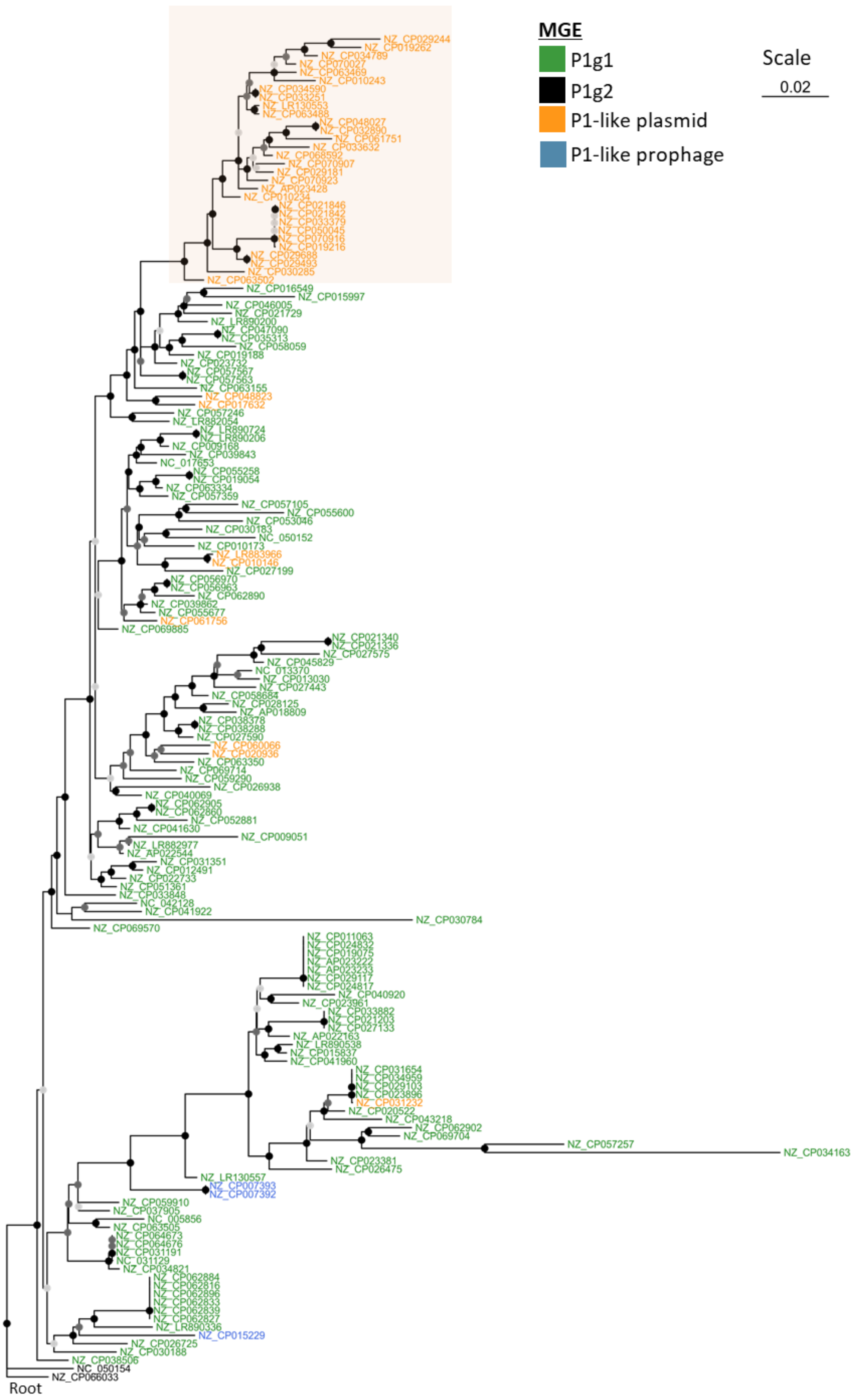

Figure S14: Phylogenetic tree of P1-like mobile elements using members of P1g2 as outgroup to root the tree. P1-like MGEs (same as in Figure 6) and two members of the P1g2 subgroup (D6[11] and NZ_CP066033) were pooled and a phylogenetically was computed based on a global alignment of 15 gene families.
