# Supplementary material for: Phage-plasmids promote recombination and emergence of phages and plasmids
Source: Nat Commun. 2024 Feb 20;15:1545. doi: 10.1038/s41467-024-45757-3 (PMC10879196; doi:10.1038/s41467-024-45757-3)
Supplement: Supplementary file 3 — Description of Additional Supplementary Files [file 41467_2024_45757_MOESM3_ESM.pdf]

Description of Additional Supplementary Files:

Supplementary Data 1: Overview of Mobile Genetic Elements Studied in This Work: Included are NCBI accession numbers, MGE type, group/cluster, and size (in base pairs).

Supplementary Data 2: Gene repertoire relatedness between MGE groups.

Supplementary Data 3: Recombining and Non-recombining genes were annotated using various databases (see Methods). Table includes information on the genome (as Supplementary Dataset S1), gene and protein identifier of the NCBI database, protein size (in aa), recombination category (RG, NRG, NRG-nh), best hits with the databases used for annotation.

Supplementary Data 4: List of recombining genes that were assigned in gene families.

Supplementary Data 5: Annotated genes, both recombining and non-recombining, were categorized into functional groups and quantified. Each group was individually subjected to contingency tables to test for enrichment in recombining genes (RGs) and non-recombining genes (NRGs) using Fisher's exact test. Difference Sum ratios were calculated (see Methods) to measure the extent of variation between these groups.

Supplementary Data 6: As Supplementary Dataset S5 but recombining genes were classed into genes that were exchanged between same (wMGE) and different (bMGE) types of mobile genetic elements. Enrichment tests were done for bMGE vs. wMGE.

Supplementary Data 7: List of P1-related MGEs (plasmids, integrated prophages and P1 as reference) and their characteristics. This includes if they are close related (and therefore used in the phylogenetic tree), their sizes (in bp), group assignment, relatedness to a P1-P-P, number of related genes to the P-P and to conserved P1g1 genes, and number of genes in each PHROG category.
