# Supplementary material for: Phage-plasmids promote recombination and emergence of phages and plasmids
Source: Nat Commun. 2024 Feb 20;15:1545. doi: 10.1038/s41467-024-45757-3 (PMC10879196; doi:10.1038/s41467-024-45757-3)
Supplement: Supplementary file 11 — Reporting Summary [file 41467_2024_45757_MOESM11_ESM.pdf]

nature portfolio

Corresponding author(s): Eugen Pfeifer (eugen.pfeifer@inrae.fr)
Eduardo P.C. Rocha (erocha@pasteur.fr)

Last updated by author(s): Jan 11, 2024

Reporting Summary

Nature Portfolio wishes to improve the reproducibility of the work that we publish. This form provides structure for consistency and transparency in reporting. For further information on Nature Portfolio policies, see our Editorial Policies and the Editorial Policy Checklist.

Statistics

For all statistical analyses, confirm that the following items are present in the figure legend, table legend, main text, or Methods section.

n/a	Confirmed	
[ ]	[x]	The exact sample size (n) for each experimental group/condition, given as a discrete number and unit of measurement
[x]	[ ]	A statement on whether measurements were taken from distinct samples or whether the same sample was measured repeatedly
[ ]	[x]	The statistical test(s) used AND whether they are one- or two-sided<br>*Only common tests should be described solely by name; describe more complex techniques in the Methods section.*
[x]	[ ]	A description of all covariates tested
[ ]	[x]	A description of any assumptions or corrections, such as tests of normality and adjustment for multiple comparisons
[ ]	[x]	A full description of the statistical parameters including central tendency (e.g. means) or other basic estimates (e.g. regression coefficient) AND variation (e.g. standard deviation) or associated estimates of uncertainty (e.g. confidence intervals)
[ ]	[x]	For null hypothesis testing, the test statistic (e.g. F, t, r) with confidence intervals, effect sizes, degrees of freedom and P value noted<br>*Give P values as exact values whenever suitable.*
[x]	[ ]	For Bayesian analysis, information on the choice of priors and Markov chain Monte Carlo settings
[x]	[ ]	For hierarchical and complex designs, identification of the appropriate level for tests and full reporting of outcomes
[x]	[ ]	Estimates of effect sizes (e.g. Cohen's d, Pearson's r), indicating how they were calculated

Our web collection on statistics for biologists contains articles on many of the points above.

Software and code

Policy information about availability of computer code

Data collection	Genomes of 1416 phage-plasmids, 3585 phages and 20274 plasmids were downloaded from the non-redundant NCBI RefSeq database (http://www.ncbi.nlm.nih.gov/RefSeq/) in March, 2021. All reference IDs (n= 25275) are listed in Table S1.
Data analysis	- Prophage regions were predicted using VirSorter2 (v.2.2.3)<br>- Gene similarities were computed with MMseqs2 (v. 13-45111)<br>- Gene repertoire relatedness was computed using a customized R script that was made available (see code availability section)<br>- Phages were grouped by vConTACT2 (v. 0.9.19) and plasmids with COPLA v. 1.0<br>- Gene functions were annotated using HMMER v. 3.3.2 with profiles from different databases: PHROGs (September 2021), DefenseFinder v. 1.0.9, AMRFinderPlus v. 3.10.18, or with MMseqs2 in the case of VFDB (last accessed on 17.12.2022)<br>- Pangenome analysis was done using PanACoTA v.1.2 and tree calculation with IQ-TREE v. 2.0.6<br>- In the customized R scripts, following packages were used for the data analysis:<br>data.table 1.14.10, tidyverse 2.0.0, seqinr 4.2 – 23, readxl 1.4.3, RColorBrewer 1.1, igraph 1.6, gggenomes 0.9.5.9, ggtree 3.2.1

For manuscripts utilizing custom algorithms or software that are central to the research but not yet described in published literature, software must be made available to editors and reviewers. We strongly encourage code deposition in a community repository (e.g. GitHub). See the Nature Portfolio guidelines for submitting code & software for further information.

Data

Policy information about availability of data

All manuscripts must include a data availability statement. This statement should provide the following information, where applicable:

- Accession codes, unique identifiers, or web links for publicly available datasets
- A description of any restrictions on data availability
- For clinical datasets or third party data, please ensure that the statement adheres to our policy

The genomics data employed in this study is openly accessible and can be obtained from the NCBI database (https://www.ncbi.nlm.nih.gov/) using the respective gene, protein, or genome IDs. All accession numbers for these genomes are provided in Table S1. The assignments of recombining genes generated in this study have been deposited in a Figshare repository.

Research involving human participants, their data, or biological material

Policy information about studies with human participants or human data. See also policy information about sex, gender (identity/presentation), and sexual orientation and race, ethnicity and racism.

Reporting on sex and gender	N/A
Reporting on race, ethnicity, or other socially relevant groupings	N/A
Population characteristics	N/A
Recruitment	N/A
Ethics oversight	N/A

Note that full information on the approval of the study protocol must also be provided in the manuscript.

Field-specific reporting

Please select the one below that is the best fit for your research. If you are not sure, read the appropriate sections before making your selection.

☒ Life sciences ☐ Behavioural & social sciences ☐ Ecological, evolutionary & environmental sciences

For a reference copy of the document with all sections, see nature.com/documents/nr-reporting-summary-flat.pdf

Life sciences study design

All studies must disclose on these points even when the disclosure is negative.

Sample size	In this study, 1416 phage-plasmids, 3585 phages and 20274 plasmids were studied. Their genomes were downloaded from the non-redundant NCBI RefSeq database (http://www.ncbi.nlm.nih.gov/RefSeq/) and all accession numbers are stored in table S1.
Data exclusions	In the quantification of recombining genes per mobile genetic element (Figure 2), it is noteworthy that not all phages and plasmids exhibit both recombining and non-recombining genes. Specifically, the subset comprising 110 phages and 217 plasmids, which lacked both recombining and non-recombining genes, was deliberately excluded from this analysis as well as all subsequent procedures. This exclusion was carried out with a specific focus on the study's emphasis on gene flow between recombining elements and the potential conversion between related cases. These cases were omitted due to their absence of homologous sequences with other elements.
Replication	In this study, no experimental validation was undertaken, implying that there was no necessity for biological or technical replications. All data presented in this study can be reproduced using the provided datasets, following the outlined methods in the Methods section, and referring to the information provided in the Data and Code Availability sections.
Randomization	Our study did not involve any biological experiments or clinical trials, rendering randomization of samples unnecessary as an experimental control.
Blinding	In our study, blinding was not applied. Given that our research primarily involved genomic datasets of mobile genetic elements and focused on in silico analyses to examine their recombination and conversion dynamics, the absence of biological experiments rendered blinding unnecessary. Furthermore, our study, did not involve direct sample handling by humans or subjective assessments, naturally reduced the potential for biases that blinding typically addresses in experiments.

Reporting for specific materials, systems and methods

We require information from authors about some types of materials, experimental systems and methods used in many studies. Here, indicate whether each material, system or method listed is relevant to your study. If you are not sure if a list item applies to your research, read the appropriate section before selecting a response.

Materials & experimental systems

n/a	Involved in the study
☒	☐ Antibodies
☒	☐ Eukaryotic cell lines
☒	☐ Palaeontology and archaeology
☒	☐ Animals and other organisms
☒	☐ Clinical data
☒	☐ Dual use research of concern
☒	☐ Plants

Methods

n/a	Involved in the study
☒	☐ ChIP-seq
☒	☐ Flow cytometry
☒	☐ MRI-based neuroimaging

Plants

Seed stocks	*Report on the source of all seed stocks or other plant material used. If applicable, state the seed stock centre and catalogue number. If plant specimens were collected from the field, describe the collection location, date and sampling procedures.*
Novel plant genotypes	*Describe the methods by which all novel plant genotypes were produced. This includes those generated by transgenic approaches, gene editing, chemical/radiation-based mutagenesis and hybridization. For transgenic lines, describe the transformation method, the number of independent lines analyzed and the generation upon which experiments were performed. For gene-edited lines, describe the editor used, the endogenous sequence targeted for editing, the targeting guide RNA sequence (if applicable) and how the editor was applied.*
Authentication	*Describe any authentication procedures for each seed stock used or novel genotype generated. Describe any experiments used to assess the effect of a mutation and, where applicable, how potential secondary effects (e.g. second site T-DNA insertions, mosiacism, off-target gene editing) were examined.*
